# Delivery of Dihydroergotamine Mesylate to the Upper Nasal Space for the Acute Treatment of Migraine: Technology in Action

**DOI:** 10.1089/jamp.2022.0005

**Published:** 2022-12-13

**Authors:** Wade Cooper, Sutapa Ray, Sheena K. Aurora, Stephen B. Shrewsbury, Christopher Fuller, Greg Davies, John Hoekman

**Affiliations:** ^1^ Headache and Neuropathic Pain Program, University of Michigan, Ann Arbor, Michigan, USA.; ^2^ Impel Pharmaceuticals, Seattle, Washington, USA.

**Keywords:** acute treatment, dihydroergotamine, migraine, nasal delivery, Precision Olfactory Delivery, technology

## Abstract

Oral tablets account for the majority of medications used to acutely treat migraine, but relief can be limited by their rates of dissolution and absorption. The nose is an attractive alternative route of drug delivery since it provides patient convenience of at-home use, gastrointestinal (GI) avoidance, and rapid absorption of drugs into systemic circulation because of its large surface area. However, the site of drug deposition within the nasal cavity should be considered since it can influence drug absorption. Traditional nasal devices have been shown to target drug delivery to the lower nasal space where epithelium is not best-suited for drug absorption and where there is an increased likelihood of drug clearance due to nasal drip, swallowing, or mucociliary clearance, potentially resulting in variable absorption and suboptimal efficacy. Alternatively, the upper nasal space (UNS) offers a permeable, richly vascularized epithelium with a decreased likelihood of drug loss or clearance due to the anatomy of this area. Traditional nasal pumps deposit <5% of active drug into the UNS because of the nasal cavity's complex architecture. A new technology, Precision Olfactory Delivery (POD^®^), is a handheld, manually actuated, propellant-powered, administration device that delivers drug specifically to the UNS. A dihydroergotamine (DHE) mesylate product, INP104, utilizes POD technology to deliver drug to the UNS for the acute treatment of migraine. Results from clinical studies of INP104 demonstrate a favorable pharmacokinetic profile, consistent and predictable dosing, rapid systemic levels known to be effective (similar to other DHE mesylate clinical programs), safety and tolerability on the upper nasal mucosa, and high patient acceptance. POD technology may have the potential to overcome the limitations of traditional nasal delivery systems, while utilizing the nasal delivery benefits of GI tract avoidance, rapid onset, patient convenience, and ease of use.

## Introduction

Many diseases have a chronic course and require long-term treatment, but others are episodic and acutely discomforting, and maximal benefit is required quickly from administered therapies. Pharmacological therapies need to cross from the external environment in which they are found or created into the internal environment within the human body. To achieve this, they first need to cross an epithelium—the gut most often, following the route taken by food-derived nutrients. Other routes include bridging the protective tegument of the body—the skin, or following the path of other vital molecules, via the airways.^(1–3)^ It has been recognized for some time that inhalation offers an opportunity to bypass the first line of defense—the hairs, mucus, and mucociliary clearance in the nose—and allows volatile molecules access to the pulmonary epithelium across which oxygen and carbon dioxide are exchanged.^(4–6)^

However, consistent, reliable delivery of pharmacological agents to the alveoli of the lungs has been met with challenges.^(7–9)^ Delivering drugs through the nose is another option, but this has often involved the delivery of a cloud of droplets to the lower nasal space or vestibule, and formulations that deposit there need to be absorbed rapidly before gravity leads to droplets coalescing and dripping out of the nostril or back into the nasopharynx.^(1,9–11)^ Particles can also get trapped in the mucus and cleared (and then swallowed) by mucociliary clearance—another of the efficient protective mechanisms that need to be overcome by therapeutics.^(4,12)^ The upper nasal space (UNS), however, is an underutilized route of administration.^(9)^ In this space, the olfactory epithelium has nonmotile ciliated cells, is richly vascularized, and has increased permeability compared with the lower nasal space, making it an ideal route for rapid drug absorption into the systemic circulation, while simultaneously avoiding delayed absorption from the gastrointestinal (GI) tract or extensive hepatic first-pass metabolism in the liver.^(3,9,12–15)^ In addition, deposition into the pulmonary airways is largely avoided since large drug particles generally deposit in the upper airways.^(16)^ This article describes a technology that has been specifically designed and developed to deliver therapies to the previously underutilized UNS, for situations when rapid and extensive absorption are required for therapeutic benefit.

## Optimizing Nasal Delivery of Migraine Therapies

### Challenges with oral drug delivery for migraine

Migraine is a debilitating, recurrent headache disorder with a high prevalence and disease burden.^(17,18)^ Recent epidemiological studies revealed migraine to be among the top leading causes of disability globally—in fact, it is the second most common cause worldwide and the most common for young women.^(19,20)^ Migraine is a variable and complex disorder, characterized by a broad spectrum of symptoms associated with neurological, vascular, autonomic, and GI dysfunction.^(21–24)^ Migraine attacks are phasic, where symptoms may be present before initiation of a migraine, during a migraine, or in between migraine attacks.^(21,22,24,25)^ The pathophysiology of migraine results from changes to multiple central and peripheral nervous system pathways, including alterations to thalamocortical connectivity, cortical hyperexcitability, changes in the release of neuropeptides such as calcitonin gene-related peptide, alterations within the brainstem, and sensitization of the trigeminovascular system (including dysregulated serotonergic, adrenergic, and dopaminergic signaling).^(22,24,26–28)^ Because of the multifactorial nature of migraine pathophysiology, drug targets with broader pharmacology may be better suited for full-spectrum symptom relief, particularly for those patients with an incomplete, inconsistent, or lack of response to agents with more narrow pharmacological range.^(21,22,24,29)^

Oral medications account for over 90% of therapies utilized to treat migraine and include triptans, gepants, ditans, nonsteroidal anti-inflammatory drugs, simple analgesics (acetaminophen), and opioids, which are not recommended.^(30–35)^ However, oral delivery of some acute therapies for migraine can be associated with intra or inter-patient variability in drug absorption, which can be further complicated by GI symptoms often associated with migraine, such as nausea, vomiting, and delayed gastric emptying.^(1,8,23,36,37)^ Furthermore, the bioavailability (i.e., the extent to which a drug reaches the systemic circulation) and the time to maximum plasma concentration (*T*_max_) of oral medications may be affected by recent or concurrent consumption of food.^(38–40)^ One study reported that consuming an oral migraine drug with a high-fat meal may delay the median time to reach *T*_max_ by ∼1 hour.^(41)^

There are several reasons for needing an alternative to oral migraine medications, such as:

- patients who have difficulty swallowing oral medications^(42,43)^
- patients who have nausea and vomiting associated with their migraine, which may lead to an aversion to anything taken by mouth or drug loss in vomitus^(23,24,44–46)^
- patients who are unwilling to take an oral medication because of GI complications^(1,46)^
- patients who have other GI comorbidities or autonomic dysfunction that may affect drug absorption^(1,23,46)^
- patients who cycle through multiple oral therapies without finding relief^(23)^

Therefore, consideration of reliable, well-tolerated non-oral routes of administration for acute therapies for migraine is recommended by the current American Headache Society guidelines.^(47)^ Administration of acute therapies by injection is an alternative, such as sumatriptan delivered subcutaneously, which may be preferable for those who experience nausea and vomiting and has been reported to quickly alleviate acute symptoms of migraine.^(48,49)^ Yet, subcutaneously administered sumatriptan has been associated with increased adverse events (AEs) and lower patient convenience for some, and it may not be suitable for patients who have needle phobia.^(46,49)^

### Nasal delivery of acute treatments for migraine

The site of drug deposition within the nose is an underappreciated feature of nasal drug delivery, as the nasal cavity can be divided into an upper and lower nasal space, which differ in epithelia type, mucociliary function, and vascular supply.^(1,12,14,50–54)^ A recently published review by Martin et al. provides a detailed and illustrated overview of the upper and lower nasal space.^(52)^ Briefly, the lower nasal space is located anteriorly, just posterior to the nostrils, and includes structures such as the vestibule and the nasal turbinates.^(52–54)^ The vestibule is lined with a non-ciliated squamous epithelium that is poorly suited for drug absorption, and although the turbinates are covered by more absorptive pseudostratified cuboidal-columnar respiratory epithelium, it is covered with highly motile cilia and variable amounts of mucus. Most nasal sprays have been shown to target the lower nasal space because they were developed to treat local nasal diseases, where systemic absorption was neither wanted nor required.^(1,9,10,52,53)^

Additionally, drug delivery to the lower nasal space may result in significant drug loss from the nose because of increased mucociliary clearance, swallowing, or expectoration (or nose blowing), which may lead to variability in drug absorption.^(1,9,12,14,52,55–57)^ Tissue architecture in the UNS may render it more suitable for systemic drug absorption.^(9,14)^ Its olfactory epithelium, consisting of pseudostratified columnar cells, is more permeable than the respiratory epithelium found in the lower nasal space.^(14,15,52,58)^ Olfactory epithelium accounts for ∼3% of the nasal surface area and contains cilia, which lack the dynein arms linking microtubules that are required for motility. These nonmotile cilia increase the surface area for odorant reception, and their immotile nature may contribute to the slower mucociliary clearance in the olfactory region,^(12,52,53,56)^ which could further increase drug absorption.

Mucus covering the olfactory epithelium is produced and secreted by Bowman's glands, and animal studies have demonstrated that mucus turnover in the olfactory region may take several days compared with 10 minutes in the respiratory region, which is predominantly covered in motile cilia.^(6,12,52,55–57)^ Recent work utilizing a computer simulation model to predict drug absorption within the nasal cavity demonstrated that particles deposited into the posterior region, which included the olfactory region, were more readily absorbed compared with those deposited more anteriorly in the nasal vestibule.^(59)^ Together, these attributes may lead to more consistent and predictable absorption when drugs are targeted to the UNS.^(1,9,14,52)^

Delivery to the UNS may be of particular interest in individuals with migraine because of the presence of autonomic dysfunction within the nose during an acute attack. The nasal congestion and rhinorrhea often associated with migraine can be caused by autonomic dysfunction.^(60,61)^ In fact, studies have demonstrated that individuals who self-identified with a sinus headache were more likely to have migraine when formally evaluated by the International Headache Society criteria and they frequently experienced nasal congestion or rhinorrhea as part of their headache experience.^(61,62)^ Furthermore, rhinitis and other nasal symptoms are associated with migraine, which may be due to shared pathophysiology of the trigeminal autonomic system, and can be accompanied by edema within the nasal cavities.^(62–65)^ Considering that the lower nasal space may have more inconsistent drug absorption due to the factors discussed above (e.g., mucociliary clearance, drug swallowing or loss from dripping),^(1,9,12,14,52,55–57)^ it is possible that drug absorption within the lower nasal space is more erratic in the context of sinus symptoms. Theoretically, depositing drug within the UNS could result in more consistent drug delivery even during bouts of allergy, congestion, or migraine-associated rhinitis.

Several products available for the acute treatment of migraine are traditional nasal sprays.^(9,10,66–69)^ Despite their challenges, traditional nasal sprays have advantages for patients with severe nausea and vomiting or difficulty swallowing oral medications, or for patients who have not responded to standard oral treatments and may appropriately be prescribed a non-oral option.^(32,47,70,71)^ The addition of hydrogels (chitosan, hydroxypropyl methylcellulose, etc.) or permeation-enhancing excipients to these lower nasal space-targeted formulations can improve mucoadhesion and bioavailability.^(12)^ There is an available nasal product that does deliver some drug to the UNS for the acute treatment of migraine: ONZETRA^®^ Xsail^®^ (Currax Pharmaceuticals LLC, Morristown, NJ) delivers sumatriptan powder via breath-powered expulsion to the UNS. It houses sumatriptan powder within a disposable nosepiece designed for single use in a hypromellose capsule.^(72,73)^ The capsule is attached to a reusable delivery device, which includes a mouthpiece and piercing mechanism to access the capsule.^(73)^ However, drug delivery into the nostril requires blowing through a mouthpiece while simultaneously inserting the nosepiece into a nostril, which requires breathing coordination from a conscious patient for proper drug delivery.^(72–74)^ Lastly, triptans generally have a narrow optimal time window during a migraine attack for effective medication administration along with a shorter receptor occupancy time, and thus, in some patients, provide inadequate sustained pain relief.^(75–81)^

### Nasal delivery of dihydroergotamine mesylate for acute treatment of migraine

Dihydroergotamine (DHE) acts broadly on several serotonergic, dopaminergic, and adrenergic receptors, and has been shown to inhibit the trigeminal pathway and reverse neuronal sensitization associated with migraine attacks.^(70,82–84)^ DHE has a long half-life with biphasic elimination and has been shown to slowly dissociate from receptors, which may underlie its proven benefit in recurrent headache and multiple forms of difficult-to-treat migraine (e.g., menstrual migraine, medication overuse headache, triptan-resistant migraine, and severe or prolonged migraine).^(9,29,70,71,76,85,86)^ Intravenous (IV) administration of DHE mesylate has a long and reputable history as an acute treatment of migraine because of its rapid onset. Yet frequent AEs, such as nausea and vomiting (due to high maximum plasma concentration with IV injection), and the inconvenience of IV administration in a doctor's office or hospital are shortcomings that may limit its use, prompting the development of alternative delivery methods.

Further, patients generally express a preference for needle-free options, which can include a variety of oral and non-oral delivery methods.^(87,88)^ Oral formulations of DHE mesylate are not used in the United States because of poor bioavailability (<1%).^(9,86,89,90)^ Rectal suppository DHE mesylate is available only by custom compounding and is limited by availability, variable absorption, and patient preference.^(91–94)^ MAP0004 (LEVADEX™; MAP Pharmaceuticals, Mountain View, CA) was a DHE mesylate product developed as an orally inhaled suspension in a hydrofluoroalkane (HFA) propellant that completed a comprehensive clinical development program, demonstrating efficacy and tolerability. In clinical studies, MAP0004 provided pain relief for some patients with severe pain as early as 10 minutes (5% [9/166] compared to 1% [2/187] on placebo; *p* = 0.0242), with a time to pain freedom of 23 minutes (*p* = 0.0203) overall compared with placebo.^(95)^

Although it was an eagerly awaited product, its market approval was rejected by the Food and Drug Administration (FDA) and its clinical development was terminated based on manufacturing concerns, primarily challenges with consistent dose delivery.^(7,9)^ MIGRANAL^®^ (Bausch Health Companies, Inc., or its affiliates, Bridgewater, NJ) and two generic versions are the only approved DHE mesylate nasal sprays, which primarily reach the lower nasal space.^(66,96,97)^ MIGRANAL has been associated with problems of drug spillage out of the nose or into the nasopharynx, which may explain its reduced bioavailability, variable therapeutic efficacy, and associated reports of adverse taste (8%) and nausea (10%) in some patients.^(9,66,90)^ These observations are in line with a majority of traditional nasal products that spray a cloud of drug inside the nostril, where much of the drug product may be lost because of swallowing or dripping out of the nostril.^(9,14,51)^ Additionally, the dosing of MIGRANAL may be challenging since patients must deliver 1 spray in each nostril, followed by a subsequent spray in each nostril after waiting 15 minutes.^(66)^

## Precision Olfactory Delivery (POD^®^) Technology

The Precision Olfactory Delivery (POD) nasal drug delivery platform (Impel Pharmaceuticals, Seattle, WA) utilizes the UNS to improve drug bioavailability and provide consistent, rapid, and reliable absorption of drug. This section will describe the challenge of targeting drug delivery to the UNS and how the POD technology overcomes this along with the versatility, efficiency, convenience, and patient-centricity of the POD technology. Standard nasal devices deliver <5% of active drug to the UNS.^(9,14,51)^ To reach the UNS, drugs must first pass the flow-limiting, narrow opening of the nasal valve, which is bordered by the upper lateral cartilage, septum, and head of the inferior turbinate, and then navigate through the nasal turbinates before their deposition at the pinnacle of the nasal cavity.^(14,98)^

**FIG. 1.**
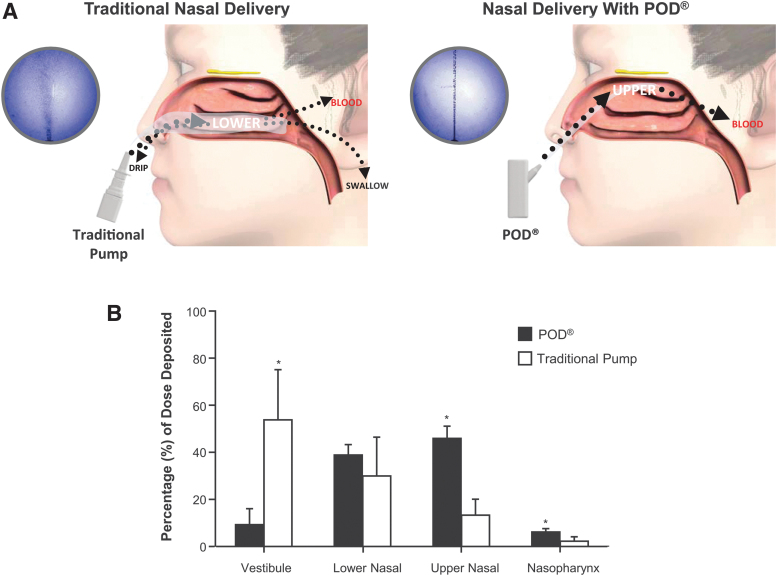
Traditional Nasal Delivery versus POD^®^. **(A)** High speed laser imaging of the emitted aerosol plumes from MIGRANAL (left image) and the POD device (right image) are depicted in the purple circles. Traditional nasal pumps have been shown to deliver drug to the lower nasal space via a widespread puff, where there is an increased likelihood of drug loss due to dripping from the nose or swallowing.^(9,52)^ POD technology delivers a narrowly targeted plume to the UNS, where the drug is less likely to be lost and can be absorbed by its highly vascularized and permeable epithelium.^(9,13–15,52)^ **(B)** Quantification of nasal deposition by SPECT imaging revealed that the traditional nasal pump deposited a majority of its dose into the vestibular region in the anterior nasal cavity and that the POD device provided significantly increased deposition in the UNS compared with the traditional nasal pump.^(9)^ *p < 0.05 Student's *t*-test. POD, Precision Olfactory Technology; SPECT, single photon emission computed tomography; UNS, upper nasal space.

Devices that utilize POD technology are handheld, manually actuated, and gas- or liquid-propellant-powered, and designed to gently deliver a narrow, focused stream-like plume of liquid (e.g., DHE mesylate, INP104) or powder formulations (e.g., INP105, olanzapine) to the UNS.^(14,90,99)^ A precise quantity of drug is consistently delivered to the same target with each use, with highly reproducible volume, velocity, and spray angle. A proprietary nozzle design for liquid DHE mesylate (in the case of INP104), with specific modifications for other programs, allows for the formation of a narrow plume of drug droplets to effectively pass through the nasal valve to reach the rich vasculature of the UNS. In addition, a subsequent stream of propellant from the biphasic emission pushes drug deeper into the UNS and maximizes drug dispersion over the superior turbinate and olfactory region of the UNS.^(14)^ Drug deposition within the nasal cavity was determined for POD compared with a traditional nasal pump, demonstrating that a majority of study dose was deposited in the anterior lower nasal cavity with the traditional cloud delivery of the nasal pump. In contrast, use of the POD device resulted in an increased deposition in the UNS (Fig. 1).^(9)^

**FIG. 2.**
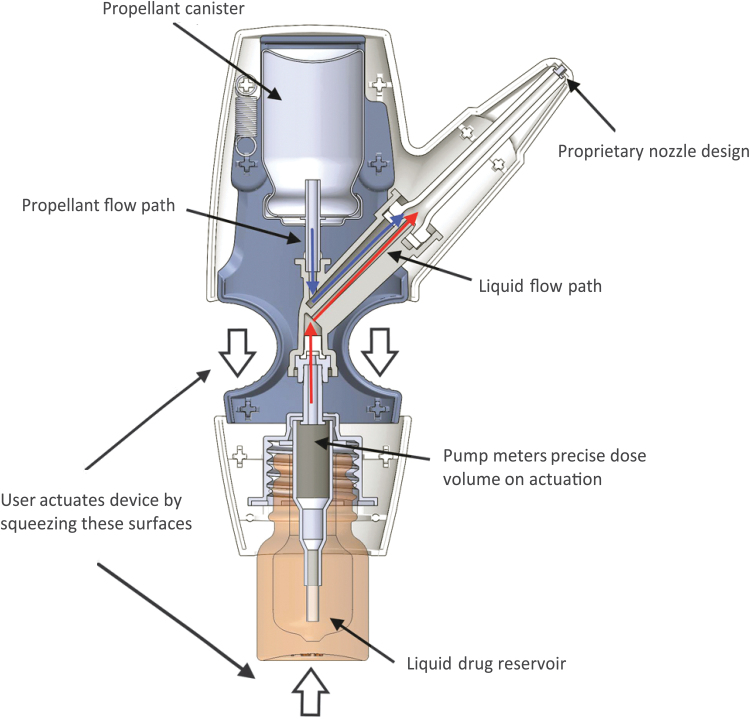
Diagram of the INP104 product. Delivery of DHE mesylate using POD technology (INP104) involves manual actuation by the user (squeezing of the surfaces noted by the white arrows), which after priming the device draws up a precise volume of DHE mesylate (red arrows) from the liquid drug reservoir to the dose chamber. The propellant, HFA-134a (blue arrows), is directed from the propellant canister, and its release creates a pressure as the gas expands, channeling DHE mesylate through the nozzle and expelling the drug from the device and to the UNS. Subsequently, the HFA-134a propellant pushes the released DHE mesylate deeper into the UNS in a second phase of deposition. DHE, dihydroergotamine; HFA, hydrofluoroalkane; POD, Precision Olfactory Delivery.

Drugs used with the POD technology may be formulated as powder, liquid, or other suitable nasal delivery forms before mixing with the propellant at the time of use. Versatility in drug formulation allows for the potential to utilize POD technology for the delivery of other liquid or dry powder small molecules and larger molecules, including biologics. Its propellant-powered delivery means that breath coordination, breath hold, or active sniff is not necessary. A patient can be unconscious and still receive medication since drug delivery can be administered by a caregiver or a medical professional. In all iterations of POD programs, the drug product and propellant do not come into contact with each other before manual actuation. This results in a biphasic emission through the POD device, wherein the propellant launches the drug into the UNS and then pushes it deep into the UNS, spreading it across the highly absorptive epithelium (see further details below, Fig. 2).

The propellant only contacts the drug droplets (liquid formulations or particles [powder formulations]) for a very brief period (less than a second), limiting the contact time and preventing the relatively insoluble, amorphous drug particles from dissolving in the HFA propellant. By maintaining separation between the propellant and the liquid or powder drug until their delivery, the POD also overcomes some of the manufacturing issues encountered when the drug is suspended in propellant (such as dose uniformity and drug stability), especially for extended periods of time.^(14)^ Various propellants can be used (e.g., liquid, gas) with the POD; examples of suitable propellants include HFAs or nitrogen.

**FIG. 3.**
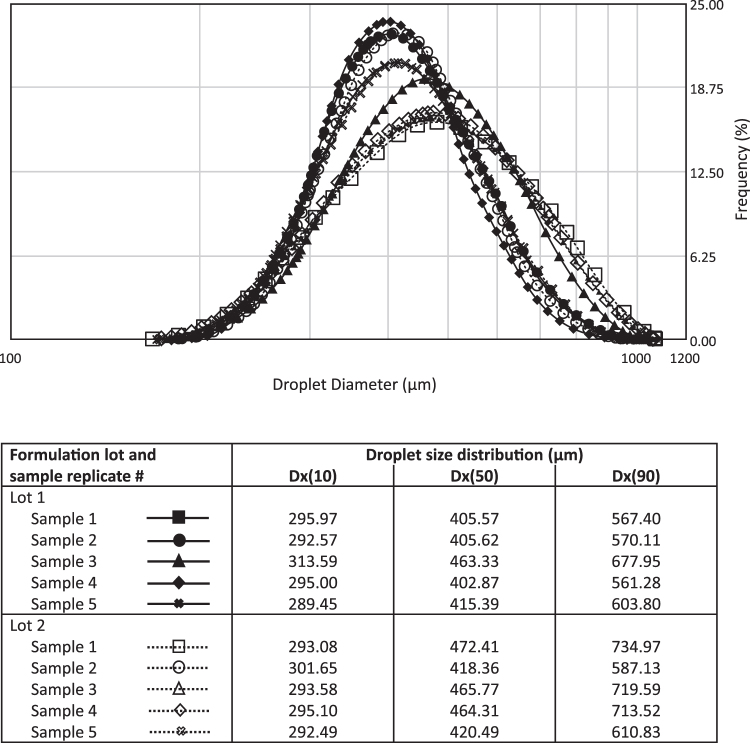
Droplet size distribution profile of INP104. The droplet size distribution profile was assessed for multiple lots of INP104 using Spraytec technology, confirming that INP104 delivers a consistent spray of large droplets (∼289–735 μm) well outside the respirable range, regardless of the orientation of the device. Comparable droplet size distribution profiles were obtained when the device was stored in either the upright or inverted position. Dx(10), Dx(50), and Dx(90) values denote the size of particles wherein 10%, 50%, or 90% of all particles, respectively, are found.

The dose uniformity and consistency achieved using POD delivery within the UNS is also influenced by the size distribution of the droplets themselves. The POD system was developed to deliver large drug particles specifically to the UNS, with a minimal fraction present in the respirable range. Typically, particles or droplets smaller than 5 microns are considered to be within the respirable range, while larger particles are generally deposited within the upper airways and mouth.^(100)^ Using Spraytec methodology, the droplet size distribution profile of INP104 was found to be consistent across multiple formulation lots and POD storage orientations (either upright or inverted; Fig. 3). Andersen Cascade Impactor data further support that upon actuation, a negligible fraction of droplets can be detected within the respirable fraction, conservatively defined here as <9 μm (Table 1). Collectively, these data support that the POD design consistently delivers larger droplets beyond the respirable range to the UNS, contributing to uniform drug delivery.

**Table 1.** Andersen Cascade Impactor Findings for INP104

Lot	Device storage orientation	Parameter	Result
1	Upright	% <9 μm	0.30%
		Total recovery (%LC)	97.18%
	Inverted	% <9 μm	0.12%
		Total recovery (%LC)	98.22%
2	Upright	% <9 μm	0.08%
		Total recovery (%LC)	99.06%
	Inverted	% <9 μm	0.10%
		Total recovery (%LC)	102.86%

The majority of droplets in INP104 as detected by ACI are outside the respirable range (conservatively defined here as 9 microns), regardless of whether the device is stored in the upright or inverted orientation.

ACI, Andersen Cascade Impactor; LC, label claim.

The POD technology in the case of INP104 (TRUDHESA^®^), which was FDA approved in September 2021 for the acute treatment of migraine, utilizes a vial containing 4 mg/mL DHE mesylate liquid solution (unchanged from the formulation that has been marketed for decades) and a pressurized canister of HFA-134a propellant to deliver 1.45 mg of DHE mesylate in two sprays (Fig. 2). Single-squeeze manual actuation by the user sequentially draws a metered volume of DHE mesylate into the dose chamber, followed by release of a separate metered volume of propellant into the same dose chamber that gently propels the dose through the nozzle, out of the tip and into the nose, through the nasal valve and into the UNS. More specifically, intranasal dosing of DHE mesylate occurs via two phases, as noted above.

Initially, the HFA-134a propellant expands within the device as it converts from liquid to gas, which provides the energy to expel the metered dose of DHE mesylate from the dose chamber through the nozzle. This completes the first phase of drug delivery in which the majority of the dose is delivered. As residual gaseous HFA-134a flow continues through the device, the second phase of delivery ensues, which pushes the dose of DHE mesylate deeper into the UNS and disperses it across the surface of the UNS. The whole process from actuation to end of delivery takes approximately half a second. A single manual actuation of INP104 releases HFA-134a that is similar to the HFA exposure observed with metered-dose inhalers.

## INP104 Clinical Data in Support of POD Technology

Safety and pharmacokinetics of INP104 were assessed in the open-label, randomized, three-period, three-way crossover, Phase 1 STOP 101 study, in which healthy subjects received single doses of INP104 (1.45 mg), IV DHE mesylate (1.0 mg), and MIGRANAL (2.0 mg).^(90)^ Long-term safety, tolerability, and exploratory efficacy of intermittent INP104 use over 24 and 52 weeks were assessed in the pivotal, open-label Phase 3 STOP 301 study.^(101)^

### Rapid onset and efficient uptake into the bloodstream

A summary of INP104 pharmacokinetic parameters for all subjects in the STOP 101 safety population (i.e., all subjects who received at least one dose of any investigational product) is presented in Table 2, which includes an additional area under the curve (AUC)_0–2h_ analysis performed after publication of this study. After administration of INP104, DHE plasma levels were 93% of the mean maximum observed plasma concentration (*C*_max_) by 20 minutes, and the mean *C*_max_ of INP104 was 10-fold lower than the *C*_max_ seen with IV DHE yet >4-fold higher than the mean *C*_max_ of MIGRANAL. The mean *C*_max_ values for INP104, IV DHE mesylate, and MIGRANAL were 1301, 14,190, and 299.6 pg/mL, respectively. INP104 achieved plasma concentrations comparable to IV DHE mesylate from 30 minutes to 48 hours.

**Table 2.** Summary of Key Pharmacokinetic Parameters of INP104, Dihydroergotamine Mesylate and Precision Olfactory Delivery Technology, Among the Safety Population (*N* = 36)^(90)^

Pharmacokinetic parameters	INP104
*C*_max_, pg/mL	1301 ± 668
*C*_max_ CV (%)	51.4
AUC_0–2h_ h^*^pg/mL	1603 ± 783
AUC_0–2h_ CV (%)	48.9
AUC_0–inf_ h^*^pg/mL	6275 ± 2621
AUC_0–inf_ CV (%)	41.8
*T*_max_, hours (median [min, max])	0.50 (0.33, 2.05)
*t*_1/2_, hours	11.8 ± 2.8

Values are mean ± standard deviation calculated among the safety population (patients who received at least one dose of any study treatment).

AUC_0–2h_, area under the drug concentration-time curve from time 0 to 2 hours; AUC_0–inf_, area under the drug concentration-time curve from time 0 to infinity; *C*_max_, maximum observed plasma concentration; CV, coefficient of variation; *T*_max_, time to maximum plasma concentration; *t*_1/2_, elimination half-life.

Median *T*_max_ occurred at 0.50, 0.08 (i.e., at 5 minutes—the first timepoint measured), and 0.78 hours for INP104, IV DHE mesylate, and MIGRANAL, respectively. DHE exposure in the 2 hours after administration (AUC_0–2h_) was 1603, 3022, and 387.5 h*pg/mL for INP104, IV DHE mesylate, and MIGRANAL, respectively. The half-lives of plasma DHE were similar among all three study drugs at 11.8, 14.2, and 10.4 hours for INP104, IV DHE, and MIGRANAL, respectively. Additionally, INP104 (1.45 mg) resulted in a 58.9% absolute bioavailability compared with a lower absolute bioavailability of 15.2% with a higher dose of MIGRANAL (2.0 mg, a 38% increase in dose) despite using identical formulations. Thus, the increased bioavailability of INP104 could be attributed to the difference in drug deposition within the nasal cavity as INP104 delivers DHE mesylate 2–3 cm deeper and higher in the nasal cavity compared with MIGRANAL.^(90)^

The incidence of DHE-related nausea was 0%, 9.4%, and 2.9% for INP104, IV DHE mesylate, and MIGRANAL, respectively, despite universal pretreatment with an antiemetic. Vomiting was observed in 0%, 6.3%, and 2.9% of INP104, IV DHE mesylate, and MIGRANAL users, respectively.^(90)^ Although IV DHE mesylate is regarded as a highly efficacious migraine treatment, its higher *C*_max_ likely contributes to the higher incidence of nausea and vomiting observed.^(9,90,102)^ INP104 achieved therapeutic levels quickly and matched IV DHE mesylate levels from 30 minutes to 48 hours but avoided the *C*_max_ spike seen with IV DHE mesylate.^(90)^ Together, the results demonstrated that INP104 has a favorable pharmacokinetic profile of DHE that is comparable to the highly effective IV DHE mesylate but with a lower peak plasma concentration, a reduction that likely results in a lower incidence of associated AEs.^(90)^

Importantly, the pharmacokinetic data from the STOP 101 study was supported by exploratory efficacy data from the STOP 301 Phase 3 study, which revealed that 16.3% (42/257) of patients self-reported pain relief at 15 minutes post-INP104 for their first treated migraine attack, followed by 29.6% (76/257) at 30 minutes, 47.6% (121/254) at 1 hour, and 66.3% (167/252) at 2 hours.^(101)^ Although not marketed, data from the clinical development program of MAP0004 is frequently used as reference for all DHE mesylate development products.^(9,103)^ Migraine pain relief was reported as early as 10 minutes in some patients with severe pain (with significance at 30 minutes overall compared with placebo) in a Phase 3, placebo-controlled study of MAP0004.^(95)^ In STOP 101, INP104 delivery resulted in high plasma exposure to DHE in the first 2 hours, with values similar to MAP0004 (1603 and 1447 h*pg/mL reported for INP104 and MAP0004, respectively), and in STOP 301 pain relief as an exploratory efficacy outcome was reported with INP104 as early as 15 minutes, the first timepoint investigated.^(101,102)^

### Consistent delivery

In the Phase 1 study, INP104 demonstrated a higher and more consistent plasma DHE exposure compared with MIGRANAL—fourfold for *C*_max_ and threefold for AUC. There was less variability (coefficient of variation [CV%]) for *C*_max_ (51.4% vs. 91.8%), AUC_0–2h_ (48.9% vs. 86.2%), and AUC_0–inf_ (41.8% vs. 74.7%) associated with INP104 compared with MIGRANAL, respectively, suggesting that INP104 delivered DHE mesylate more consistently than MIGRANAL. Greater variability with MIGRANAL could be because of its delivery of DHE mesylate to the lower nasal space, where product can be lost because of spillage from the nose.^(90)^ Consistent dose delivery of DHE mesylate to the UNS may result in more consistent headache relief.

### Decreased dripping and swallowing

In the Phase 1 study with healthy participants, nasal drip after MIGRANAL use was reported by 77% of users, whereas this was reported by 32% of INP104 users with minimal training. Additionally, drug running down the back of the throat was reported by 44% of MIGRANAL users, while 32% of INP104 users reported this.^(90)^ Again, the lower incidence rates observed with INP104 are most likely attributable to INP104 targeting the UNS, where there is a decreased likelihood of higher rates of mucociliary clearance and nasal drip.^(55,57,90)^

### Patient acceptability

A usability and tolerability study was performed in human participants comparing the POD device to a conventional nasal spray device using saline (reference device). Results revealed that 67% (21/31) of participants preferred the POD to the reference device, with nearly 50% having greater confidence that the study drug dose was successfully delivered. Additionally, participants rated the POD to be superior with regard to each device's deposition of saline within the olfactory region of the nasal cavity. This echoed the related assessment for the perception of saline loss down the throat or running out of the nose. Participants were better able to prime, place, and actuate the POD compared with the reference device, and 100% of participants were able to successfully actuate the POD device on their first attempt, while 29% had difficulty with the reference device.^(9)^ A patient acceptability questionnaire that was administered during the Phase 3 study demonstrated that the majority of patients found INP104 to be easy to use (∼84%) and preferred it over their current migraine therapy. Additionally, the majority of patients reported that INP104 relieved each migraine episode more consistently compared with their previous prescriptions.^(101)^

### Nasal safety with delivery to the UNS

Of the 354 enrolled patients who received ≥1 dose of INP104 in the Phase 3 study, 162 (45.8%) reported a nasal-related treatment-emergent adverse event (TEAE) in the 24-week treatment period, none of which were serious. The most common (≥10%) nasal-related TEAEs were nasal congestion (*n* = 59, 16.7%) and upper respiratory tract infection (*n* = 38, 10.7%), and most nasal-related TEAEs were rated as mild or moderate. A single, severe, nasal-related TEAE of nasal congestion was reported over the 24-week treatment period.

Nasal endoscopies were performed to evaluate alterations in the nasal mucosa using the novel Quantitative Scoring Scale for Evaluation of the Nasal Mucosa (QSS-NM) grading system and olfactory functional changes were assessed according to the validated University of Pennsylvania Smell Identification Test (UPSIT). During the 24-week period, patients exhibited minimal (≤0.2) mean increases in the QSS-NM score from baseline at all postbaseline time points. Over 90% of patients had normal upper nasal endoscopies reported throughout the 24-week trial. A total of 23 patients experienced 26 TEAEs that were associated with nasal endoscopy findings, and the majority of endoscopy or QSS-NM score changes that were associated with nasal-related TEAEs were considered unrelated or unlikely to be related to INP104 use. Only 25 patients had an UPSIT score decrease of 5 or more points over 24 weeks, and >50% of associated TEAEs were asymptomatic. Of those who were symptomatic, the majority were transient and rated as mild. A Nasal Safety Review Committee, consisting of three independent otolaryngologists, reviewed the final nasal safety data and raised no concerns regarding the nasal safety of INP104.^(101)^

## Conclusion

Although the majority of medications taken for migraine relief are oral, they are not conducive to rapid relief since their onset is limited by their rates of dissolution and absorption and by comorbid, often unrecognized, GI conditions frequently associated with migraine. Additionally, the nausea and vomiting that often presents with migraine headaches may slow or prevent absorption of oral medications. Nasal drug delivery is a suitable alternative to oral administration, providing rapid onset of action, convenience, and ease of use for patients. However, a major limitation of standard nasal delivery pumps includes drug loss from the nose because of drip, swallowing, and increased mucociliary clearance in the lower nasal space, the site of drug deposition of most traditional nasal products. The richly vascularized UNS is an attractive target for migraine drug delivery, with its increased permeability and decreased likelihood of drug clearance.

INP104 utilizes POD technology for the acute treatment of migraine and is a handheld, manually actuated, propellant-powered combination product that delivers DHE mesylate to the UNS via a narrowly focused plume that extends past the internal nasal valve in the inferior nasal cavity. Benefits of utilizing POD technology include rapid, efficient uptake of drug into the bloodstream, decreased dripping and swallowing, consistent dosing of drug, and improved patient experience. Importantly, more consistent drug delivery may result in a more reliable clinical response. Results from clinical studies of INP104 demonstrate that the core technology of the POD system is well tolerated and highly acceptable to volunteers and patients alike. Together, these benefits may overcome the limitations of traditional nasal delivery systems for the acute treatment of migraine and other conditions where consistent, rapid, adequate blood levels of drug are required.

## Notes

Reviewed by:

Stephen Silberstein

Hui Xin Ong
